# Negative Behaviors among Healthcare Professionals: Relationship with Patient Safety Culture

**DOI:** 10.3390/healthcare7010023

**Published:** 2019-02-01

**Authors:** Diana M. Layne, Lynne S. Nemeth, Martina Mueller, Mary Martin

**Affiliations:** College of Nursing, Medical University of South Carolina, Charleston, SC 29425, USA; nemethl@musc.edu (L.S.N.); muellerm@musc.edu (M.Mu.); drmarymartin@aol.com (M.Ma)

**Keywords:** patient safety, interprofessional behavior, outcomes, negative behaviors

## Abstract

Behaviors that undermine a culture of safety within hospitals threaten overall wellbeing of healthcare workers as well as patient outcomes. Existing evidence suggests negative behaviors adversely influence patient outcomes, employee satisfaction, retention, productivity, absenteeism, and employee engagement. Our objective was to examine the presence of negative behaviors within a healthcare system and the influence of negative behaviors among healthcare workers on perceptions of patient safety culture. Using a cross-sectional design, the negative behaviors in healthcare survey (NBHC) and selected composites of the Agency for Healthcare Research and Quality (AHRQ) Hospital Survey on Patient Safety Culture (HSOPS) were combined within an electronic survey which was administered to physicians, clinical and managerial staff. Exposure to contributing factors of negative behaviors was moderately correlated with elements of HSOPS, including perceptions of teamwork within units, management response to error, and overall patient safety grade. Use of aggression and fear of retaliation were moderately correlated with HSOPS management response to error. Reducing healthcare worker exposure to contributing factors of negative behavior may result in increased perceptions of teamwork within a hospital unit, while addressing use of staff aggression and fear of retaliation potentially positively influences management response to error.

## 1. Introduction

Limited evidence exists examining the relationship of negative behaviors displayed among acute care hospital interprofessional team members, although several published studies have evaluated this phenomenon within single disciplines such as nurses or physicians [1,2]. Previous research among single disciplines supports that negative behaviors are associated with negative patient outcomes, decreased productivity, employee retention, satisfaction, engagement, and increased absenteeism [3,4]. Professional and regulatory organizations such as the American Nurses Association, American College of Healthcare Executives, and the Joint Commission have responded to dangers of negative behaviors by developing position statements [4] and implementing regulations to ensure hospitals have adequate prevention measures [5].

Negative behaviors encompass the continuum of less active, less intentional forms, such as incivility, to more active, more intentional forms, such as physical violence [6,7]. Many terms exist within the literature to describe negative behaviors, including workplace bullying, violence, aggression, abuse, hostility, sabotage, and incivility. Primary characteristics defining the distinction between terms include frequency of behavior, and intentionality [6,7,8]. Another defining characteristic includes whether the behavior is described directionally, such as between peers (lateral or horizontal) or between leader and follower (vertical), which describes incidents between leader and employee and may occur bi-directionally, most commonly from leader to employee but also possibly from employee to leader. Antecedents to negative behavior within the workplace include leader characteristics, follower characteristics, interpersonal relationships, and contextual variables within the environment [9].

Healthcare worker burden exists, evidenced by the psychological and emotional consequences of negative behaviors for the healthcare worker, including decreased self-esteem, decreased passion for the profession, depression, self-hatred, and feelings of powerlessness [10,11,12]. Additionally, negative behaviors in the healthcare work environment increase patient burden of care in the form of increased medication errors [13,14], delays in treatment [13,14], increased patient falls [13,14], and increased mortality [1,15,16,17,18]. Despite extensive available literature related to patient safety, limited empirical data exist examining the relationship between negative behaviors and patient safety culture [1,4,14,19,20]. The objective of this study was to examine the presence of negative behaviors among interprofessional team members using the negative behaviors in healthcare survey (NBHC) within an acute care hospital system and evaluate how these negative behaviors impact patient safety culture, measured by the Hospital Survey on Patient Safety Culture (HSOPS). The hypothesis for this study is that the presence of negative behaviors is associated with decreased perceptions of patient safety culture.

## 2. Conceptual Framework

This study applied the antecedents and consequences of leader member exchange (LMX) framework. Dulebohn, Bommer, Liden, Brouer, and Ferris [9] theorized the complex relationship between factors influencing interpersonal interactions among team members with their leaders, environment, and each other. This framework provides a lens for healthcare leaders to adequately assess and understand behaviors that undermine a culture of safety. LMX includes three key principles, including: (1) Relationships between leaders and followers are always ever-changing relationships; (2) varying interactions between leaders and followers influence the relationships; and (3) followers develop unique relationships with leaders [9]. LMX relationships are categorized into four antecedents, including follower characteristics, leader characteristics, interpersonal relationships, and contextual variables, and defines outcomes as consequences [9].

The NBHC and HSOPS instruments were selected based on outstanding validity and reliability and use within other published studies [24]. Seven composites within the HSOPS instrument were identified and 26 total items selected with the potential to be influenced by the items measured by the NBHC instrument. Selected HSOPS composites for inclusion display acceptable psychometric properties with the exception of staffing (Cronbach alpha = 0.62), and the decision to include this composite was due to the established link of staffing and turnover intention within negative behavior literature [22]. LMX constructs align with included HSOPS composites. The NBHC subscales on use of aggression and fear of retaliation align with follower characteristics of the LMX framework, while three HSOPS composites align with leader characteristics of the LMX framework, including: Supervisor/manager expectations and actions promoting patient safety, management support for patient safety, and nonpunitive response to errors. Three HSOPS composites and one NBHC subscale align within interpersonal relationships. Teamwork within units, communication openness, teamwork across units and NBHC subscale frequency of aggression align with LMX construct of interpersonal relationships. Finally, the HSOP composite of staffing aligns with contextual variables; also included within this LMX construct are NBHC contributing factors and seriousness subscales. HSOPS patient safety grade aligns with consequences within the LMX framework. Composites not included within the study were duplicative or specifically addressed process measures of patient safety culture, which is beyond the scope of this study. Composite measures excluded were organizational learning related to patient safety, frequency of event reporting, overall perceptions of patient safety, which is addressed by overall patient safety grade, feedback and communication about error, handoffs and transitions addressed with teamwork among units, and number of reported patient safety events.

## 3. Materials and Methods

### 3.1. Study Design

A descriptive correlational cross-sectional study design was used to measure the prevalence of negative behaviors as well as participant perceptions of patient safety culture. An anonymous survey was distributed via electronic mail to all clinical employees of a North Carolina-based healthcare system. Data collection occurred over four weeks (August to September 2018) to ascertain employee perceptions of patient safety culture, and negative behaviors using valid and reliable instruments.

### 3.2. Study Setting and Participant Recruitment

Following Institutional Review Board approval from the study (UMCIRB-18-00949) site and Medical University of South Carolina, participant recruitment was undertaken in a large healthcare system. This included an academic level one trauma center and affiliated community hospitals within the Southeastern United States. An email invitation and survey link were sent to all healthcare workers currently employed within the study site hospitals. During recruitment, 12,500 individuals were emailed an invitation to participate and 527 staff members participated in the study, resulting in an overall 4.2% response rate. A power analysis prior to the initiation of the study indicated a minimum of 400 responses were required to estimate outcome proportions with precision ±5% margin of error and 95% confidence interval with a standard deviation of 0.5 [23]. This sampling strategy was used to ensure maximum participation by putting the burden of inclusion or exclusion on the principal investigator (PI) in lieu of requiring potential participants to self-select out of study participation based on their understanding of the inclusion criteria. Moreover, direct care providers along with ancillary support which may witness or endure negative behaviors were included in an attempt to capture true prevalence of negative behaviors within the study site. The PI did not directly recruit participants at the request of the healthcare system. Five research interns rounded on hospital units, and in key areas, such as the physician cafeteria with an iPad, to encourage participation. Eligible participants included individuals with direct patient care responsibilities (physicians, advanced practice professionals, nursing staff) and support services, defined as disciplines which provide services which influence patient care (pharmacy, laboratory, nutrition, nursing assistants, physical, occupational and recreational therapy, and dietary), as well as team members providing support to direct patient care providers (clinical nurse specialist, education, quality, research, and leadership roles). Excluded were student learners and employees working outside of the hospital setting, such as home health or hospice.

### 3.3. Instrument Validity and Administration

The Agency for Healthcare Research and Quality (AHRQ) Hospital Survey on Patient Safety Culture HSOPS includes 42 items grouped into 12 composites measuring patient safety and error, along with event reporting [24]. The Negative Behavior in Healthcare Survey (NBHC) is a 25-item instrument measuring contributing factors of negative behavior, seriousness of behavior, frequency of behavior, use of behavior, and fear of retaliation [21].

Specific composites within the AHRQ Hospital Survey on Patient Safety Culture HSOPS [24] instrument were selected based on their potential to be influenced by the items measured by the NBHC instrument to measure perceptions of patient safety culture. Applicable composites included teamwork within units, supervisor/manager expectations and actions promoting patient safety, management support for patient safety, communication openness, teamwork across units, staffing, and overall patient safety grade. Responses to these items included 5-point Likert type responses ranging from agree/disagree, or never/always [24]. These composites had previously demonstrated acceptable psychometric properties with the exception of staffing (Cronbach alpha = 0.62) [24]; the decision to include this composite was due to the established link of staffing and turnover intention within the negative behavior literature [22]. Composites not included within the study were duplicative or specifically addressing process measures of patient safety culture.

The NBHC instrument includes 25 items measuring contributing factors (Cronbach alpha = 0.92) and severity of negative behaviors (Cronbach alpha = 0.92), fear of retaliation (Cronbach alpha = 0.91), frequency of negative behaviors (Cronbach alpha = 0.81), and use of aggression (Cronbach alpha = 0.64) [21]. The NBHC [21] was used to measure the presence of negative behaviors by eligible participants. The NBHC instrument measures strength of agreement with behaviors which contribute to negative behavior, such as major personality clashes, power and control issues, etc., as factors that contribute to negative behavior within the work area [21], while frequency of behavior and use measure how often a behavior is witnessed or used by the study participant on a 4-point Likert scale from daily to never [21]. Seriousness measures participant perceptions of how serious negative behavior is compared to other job stressors on a 4-point Likert scale from very serious to not serious [21]. Finally, fear of retaliation measures strength of agreement with safety from retaliation when reporting negative behaviors on a 4-point Likert scale from agree strongly to disagree strongly [21]. Psychometric analysis was previously reported; results revealed convergent and divergent validity as well as internal consistency reliability with Cronbach’s alpha ranging from 0.64 to 0.92 for the five NBHC subscales [21]. The NBHC was used for this study because it was previously validated using an interprofessional sample and also included unique measures not measured elsewhere, such as use of negative behavior as well as fear of retaliation.

Study data were collected and managed using REDCap (Research Electronic Data Capture, Vanderbilt University, Nashville, TN, USA) electronic data capture tools, hosted at the Medical University of South Carolina^1^. REDCap is a secure, web-based application designed to support data capture for research studies, providing (1) an instinctual interface for validated data entry; (2) audit trails for tracing data manipulation and availability for export procedures; (3) automated export capability for seamless data downloads to common statistical packages; and (4) capability for importing data from external sources [25].

### 3.4. Data Analysis

Data were extracted from REDCap [25] and imported into SPSS 24 (IBM Corp., Armonk, NY, USA) [26] for data analysis. A consistent coding strategy was applied to the HSOPS composites based on the AHRQ Patient Safety Culture User Guide. Mean scores were calculated for each composite, with higher scores indicating positive response (neutral, agree, or strongly agree), indicating a higher degree of agreement with the specific composite, for example, teamwork within units. Additionally, data from the NBHC instrument were consistently coded where higher values indicated higher incidences of observed negative behaviors, and lower values indicated lower incidence of negative behaviors. Analysis of missing values within each HSOPS composite, as well as NBHC subscale, indicated the need to use mean value substitution to ensure sufficient sample size for data analysis. Mean substitution was used for responses with partial answers including at least two responses within a single composite or subscale. Cases were excluded if all responses were missing from all of the composites or all items within a specific composite or subscale. Assumptions for Spearman’s correlation were met. Spearman’s correlations were conducted between each of the five NBHC subscales, overall patient safety grade, and the seven HSOPS composites.

Further, due to cell size for certain categories of the HSOPS composites, Likert scale responses for both HSOPS and NBHC were collapsed into binary variables with agree or strongly agree versus neutral, disagree or strongly disagree. Binary logistic regression was used to investigate which independent variables (contributing factors, fear of retaliation, and frequency of aggression) served as predictors for HSOPS composites or overall patient safety grade. A total of 21 regression pairs were analyzed. Due to the exploratory nature of this study, *p*-values were not adjusted for multiple comparisons to protect against Type II error, i.e., false negative findings, rather than Type I. No covariates were included in the modelling due to the limited sample size [27].

## 4. Results

Overall response rate was 4% (527/12,500). The majority of respondents were female healthcare workers employed by an academic hospital with between six and thirty years of experience and between two to four years of college completed (see Table 1).

Moderate correlations greater than 0.50 [28] were identified between two of the HSOPS composites and two of the NBHC subscales, while low correlations (between 0.30 and 0.50) [28] were identified between four of the HSOPS composites and three of the NBHC subscales (see Table 2). Positive correlations existed between contributing factors and teamwork within units and response to error. Additionally, positive correlations existed between response to error, frequency of aggression, and fear of retaliation. Interpreting positive correlations indicates that as one variable increases, the other variable also increases [29]. Negative correlations existed between overall patient safety grade and exposure to contributing factors, teamwork within units and frequency of aggression, use of aggression, and fear of retaliation. Interpreting negative correlations indicates that as one variable increases, the other variable decreases [29].

### 4.1. Associations between Patient Safety Culture Based on Exposure to Negative Behaviors

#### 4.1.1. Contributing Factors

Regression results indicated that participants exposed to contributing factors significantly predicted six of the HSOPS composites. Those participants reporting less exposure to contributing factors had 8.3 higher odds of agreeing teamwork within units exists (OR = 1/0.12, χ^2^ = 62.20, *p* < 0.001). Participants reporting less exposure to contributing factors had 2.28 higher odds of also reporting disagreement management support for patient safety exists (*p* = 0.001), as well as having 2.27 higher odds reporting disagreement with communication openness (*p* = 0.02).

Additionally, participants reporting less exposure to contributing factors had about twice the odds of reporting teamwork within units than those reporting agreement with exposure to contributing factors (OR = 2.04, *p* < 0.03), of reporting inadequate staffing (OR = 2.12, *p* = 0.01), and of reporting a punitive response to error (OR = 2.17, *p* < 0.001) than those reporting a nonpunitive response to error.

#### 4.1.2. Fear of Retaliation

Fear of retaliation significantly predicted four HSOPS composites. Participants who reported experiencing fear from retaliation had almost four times higher odds to report teamwork within units than those not reporting teamwork (OR = 3.71, *p* < 0.001), and nearly two times higher odds to report agreement with supervisor/manager expectations and actions promoting patient safety than those not reporting supervisor/manager expectations and actions promoting patient safety (OR = 1.96, *p* = 0.05), while participants reporting fear of retaliation had about twice the odds of reporting inadequate staffing than those reporting less fear of retaliation (OR = 1.92, *p* = 0.01) and also had almost four times higher odds of reporting a positive response to error than those reporting a punitive response to error (OR = 3.95, *p* < 0.001).

#### 4.1.3. Frequency of Aggression

Frequency of aggression significantly predicted two HSOPS composites. Participants reporting less frequency of negative behaviors had 8.3 higher odds of reporting teamwork within units compared to those who reported no teamwork within units (*p* < 0.001), while those reporting less frequent negative behavior had 2.6 higher odds of reporting positive supervisor/manager expectations and actions promoting patient safety than those reporting negative supervisor/manager expectations and actions promoting patient safety (*p* < 0.04). Additionally, participants reporting less frequency of aggression had 2.4 higher odds to report nonpunitive response to error than those reporting a punitive response to error (*p* = 0.01).

Models between contributing factors and significant HSOPS composites accurately predicted greater than 75% of cases, with the exception of management support for patient safety, which accurately predicted 57%. Significant models involving fear of retaliation accurately predicted 70% or better with the highest accuracy within overall patient safety grade at 99%, while significant models, including frequency of behaviors, and significant HSOPS composites accurately predicted between 70% and 75%. Odds ratios, confidence intervals, significance, and overall model R^2^ are presented in Table 3. 

### 4.2. Comparing the Prevalence of Negative Behaviors at Academic vs. Community Hospitals

Aggregate NBHC scale scores indicate higher mean subscale scores for contributing factors of negative behavior, as well as mean subscale scores for seriousness, and mean use of aggression occurred within community hospitals compared to the academic medical center (Table 4). These results indicate that team members at community hospitals reported more experience with contributing factors of negative behaviors, such as rude behavior, job stress, and inadequate staffing, as well as reporting a higher perception of seriousness of negative behaviors. Team members working in community hospitals indicated less frequency of negative behaviors, while these team members reported using negative behaviors more frequently.

## 5. Discussion

Although extant studies demonstrating the relationship between teamwork and patient safety outcomes [19] and patient safety culture related to AHRQ Patient Safety Indicators are available [20], none of those studies examine the relationship between patient safety culture, negative behaviors, and patient outcomes. Thomas and Galla [30] suggested a collaborative care model as an organizational structure with safety as a core value in addition to dedicated team training would be effective interventions in building and sustaining a culture of safety within a healthcare system.

Schwappach and Richard [31] reported the link between negative experiences of healthcare workers with speaking up or experiences with nonresponse from leadership following episodes of speaking up are strongly correlated with further episodes of withholding voice. Results from the current study complement these findings. Less exposure to contributing factors of negative behaviors among healthcare workers was positively related to teamwork within units, while an inverse relationship existed between self-reported use of aggression and management response to error. Use of aggression was negatively correlated with leader response to error, indicating an inverse relationship. Further results supported that as response to error increases, frequency of negative behaviors increases. One possible explanation for this finding includes experienced staff working with a newer leader challenging authority to maintain what may be a less optimal unit culture, or staff working with an inconsistent leader who does not always follow through on responding to error. Based on the limited available research linking exposure to negative behaviors, patient safety culture, and publicly reported patient safety outcomes, additional studies are necessary to further evaluate these relationships and potential predictors of perceptions of patient safety culture based on experiences. Consideration of incorporating a specific AHRQ HSOPS composite related to healthcare worker exposure to negative behaviors within the workplace may provide meaningful data to target future interventions.

Fear of retaliation was negatively correlated with overall patient safety grade. This finding suggests that those participants who reported being more fearful of retaliation reported a lower overall patient safety grade. Future interventions to increase patient safety culture within an organization should consider specific strategies to reduce team member fear of retaliation by management. Further, team members supporting each other by creating an environment which allows team members to communicate openly without fear of reprisal may be another critical step in reducing the prevalence of negative behaviors as well as increase patient safety culture based on these findings.

Two of the NBHC subscales had limited responses (seriousness and use of aggression); a cross-tabulation revealed that the majority of respondents who identified negative behaviors as serious also reported a higher agreement with teamwork within the unit and acceptable (excellent, very good, acceptable) overall patient safety grade. However, those participants who identified negative behaviors as serious also reported an increased disagreement with supervisor/manager expectations and actions promoting patient safety, management support for patient safety, communication openness, teamwork across units, and staffing. Participants who reported using aggression also agreed teamwork within the unit occurred, as well as an acceptable overall patient safety grade. Participants who indicated using aggression also disagreed with supervisor/manager expectations and actions promoting patient safety, management support for patient safety, communication openness, teamwork across units, staffing, and response to error.

In the wake of the Institute of Health Improvement (IHI) quadruple aim, which includes Joy in Work [32], these results suggest continued assessment of negative behaviors among healthcare workers may be helpful to hospital leaders in understanding perceptions of teamwork at the microsystem level of the hospital unit, while IHI also suggests the importance of measuring what matters in lieu of data collection for the sake of data collection [32]. A refinement of the existing instruments may be necessary to minimize nonresponse by participants. 

Participants working in community hospitals reported a greater frequency of exposure to contributing factors of negative behaviors, increased perception of seriousness of these behaviors, along with self-reporting less frequency of observing these behaviors that was less than counterparts at the academic medical center. This counterintuitive finding may be explained by underlying organizational culture and potential psychological safety that may exist in a smaller community.

### Limitations

Specific limitations for this study included self-selection bias, nonresponse bias, and sampling error, as the study sample may not be indicative of the population. Missing responses on several items for multiple respondents required mean imputation for at least one HSOPS composite or NBHC subscale. Low response rate could be attributable to third party subject recruitment rather than the authors, which was required by the study site due to the nature of the study. Moreover, the study site did not permit incentives that were initially planned (token gift card drawing) for participation. Another consideration is the possibility that the inclusion of multiple AHRQ HSOPS composites contributed to nonresponse due to the overall length of the combined instruments and demographic questions. The cross-sectional study design limits causal inference. Measuring the prevalence of negative behavior and the perception of patient safety culture simultaneously potentially introduces common method bias. Further, the limited sample size required collapsing response categories into dichotomous variables, which potentially limited the generalizability of the results. Finally, during the last portion of the data collection period, the study site was actively preparing for a potential natural disaster (hurricane), thus displacing attention from the recruitment and voluntary completion of this survey by the target population.

## 6. Conclusions

Despite existing limitations, our results add a novel finding related to the significant positive correlations identified between exposure to contributing factors of negative behavior and teamwork within units, as well as the significant negative correlation between use of aggression and management response to error. Future studies should focus on replicating these findings in other settings, as this is the first study known to the authors to correlate negative behaviors with patient safety culture. Moreover, additional studies evaluating the differences between the academic setting and the community hospital setting to further explain the reported differences as well as confirming a higher incidence of exposure to negative behaviors, increased perception of seriousness of negative behaviors, and self-reported use of negative behaviors within community hospitals versus academic medical centers. Once additional research is available providing insight into the exposure of negative behavior between academic medical centers and community hospitals, consideration to testing and development of targeted interventions to decrease negative behaviors should be given.

## Figures and Tables

**Table 1:** Demographic characteristics by hospital type.

Characteristics	Variables	Academic Medical Center (*n* = 215)	Community Hospitals (*n* = 162)	Both Hospital Types (*n* = 10)
Highest Degree (*n* = 484)	High School Degree	26%	0%	0%
	2–4 Years College	26%	29%	1%
	Graduate Degree	9%	6%	1%
	Doctoral Degree	7%	4%	0%
	Other	8%	7%	0%
Age (*n* = 330)	18–25	6%	2%	0%
	26–40	19%	13%	1%
	41–60	30%	22%	1%
	Greater than 60	3%	3%	1%
Gender (*n* = 373)	Female (*n* = 317)	47%	36%	2%
	Male (*n* = 56)	9%	5%	1%
Race (*n* = 346)	White	45%	35%	2%
	Black	10%	6%	0%
	Other	0%	0%	0%
Experience (*n* = 352)	1–5 years	13%	5%	0%
	6–15 years	17%	11%	1%
	16–30 years	17%	13%	1%
	Greater than 30 years	4%	6%	1%
Field (*n* = 344)	Med/Surg	7%	4%	0%
	Inpatient	6%	3%	0%
	Administration/Leadership	4%	3%	1%
	Ambulatory Care/Outpatient	4%	3%	1%
	OR and Procedure Areas	4%	3%	0%
	Critical Care	4%	2%	0%
	ED	1%	5%	0%
	Obstetrics and Pediatrics	3%	2%	0%
	Other	24%	16%	0%

Overall response rate was 4%.

**Table 2:** Spearman’s correlations between selected hospital survey on patient safety culture composites and negative behaviors in healthcare composites.

Selected HSOPS Composites	Contributing Factors	Seriousness	Frequency of Aggression	Uses Aggression	Fear of Retaliation
Teamwork within units	0.56 **	−0.11	−0.45 **	−0.37 *	−0.32 **
Supervisor/manager expectations and actions promoting patient safety	0.05	−0.27 *	−0.02	−0.11	−0.18 **
Management support for patient safety	0.29 **	0.05	−0.30 **	−0.38 *	−0.22 **
Communication openness	0.27 **	−0.05	−0.30 **	−0.32	−0.26 **
Teamwork between units	−0.12 *	−0.06	0.02	−0.22	0.09
Staffing	−0.23 **	−0.12	0.14	0.15	0.18 **
Response to error	0.44 **	−0.12	0.32 **	−0.53 **	0.49 **
Overall patient safety grade	−0.45 **	0.09	−0.19 *	0.39 *	−0.25 **

* *p* ≤ 0.05, ** *p* ≥ 0.01.

**Table 3:** Regression coefficients.

Characteristics	Variables	*n*	Odds Ratio	95% CI	*p*	Model *p*	Model R^2^
Teamwork within unit	Contributing factors	386	0.12	0.07, 0.22	<0.001	**<0.001**	0.23
	Fear of retaliation	289	3.71	2.11, 6.52	<0.001	**<0.001**	0.11
	Frequency of aggression	192	8.32	3.13, 22.13	<0.001	**<0.001**	0.18
Supervisor/manager expectations and actions promoting patient safety	Contributing factors	386	0.88	0.49, 1.59	0.67	0.67	0.001
	Fear of retaliation	290	0.51	0.26, 1.01	0.06	**0.05**	0.02
	Frequency of aggression	193	2.69	1.05, 6.88	0.04	0.04	0.05
Management support for patient safety	Contributing factors	380	2.28	1.49, 3.50	<0.001	**<0.001**	0.05
	Fear of retaliation	282	0.64	0.40, 1.01	0.07	0.07	0.02
	Frequency of aggression	188	1.49	0.81, 2.73	0.20	0.20	0.01
Communication openness	Contributing factors	384	2.28	1.32, 3.92	0.003	**0.002**	0.04
	Fear of retaliation	287	0.69	0.31, 1.16	0.13	0.12	0.01
	Frequency of aggression	191	1.77	0.79, 3.95	0.16	0.17	0.02
Teamwork between units	Contributing factors	378	0.49	0.26, 0.92	0.03	**0.03**	0.03
	Fear of retaliation	284	1.40	0.70, 2.83	0.35	0.34	0.01
	Frequency of aggression	190	0.49	0.19, 1.28	0.14	0.14	0.13
Staffing	Contributing factors	384	0.47	0.30, 0.76	0.002	**<0.001**	0.04
	Fear of retaliation	288	1.92	1.12, 3.32	0.02	**0.02**	0.03
	Frequency of aggression	193	0.87	0.40, 1.90	0.72	0.72	0.72
Response to error	Contributing factors	385	0.33	0.21, 0.52	<0.001	**<0.001**	0.08
	Fear of retaliation	289	3.96	2.21, 7.09	<0.001	**<0.001**	0.12
	Frequency of aggression	193	0.42	0.21, 0.81	0.01	**0.01**	0.05
Overall patient safety grade	Contributing Factors	382	3.09	0.28, 34.35	0.36	0.34	0.03
	Fear of retaliation	287	37569182.39	0	1.00	**0.03**	0.15
	Frequency of Aggression	191	25847597.49	0	1.00	0.20	0.08

Results rounded to the nearest hundredth; n represents total number of participants included within that binary regression. Bolded p-values indicate statistical significance at <0.001, or <0.05 level.

**Table 4:** Means (standard deviation) of composite scores from the negative behaviors in healthcare survey (NBHC) by hospital type.

NBHC Scale	Academic *N*	Academic M (SD)	Community *N*	Community M (SD)
NBHC contributing factors scale	202	2.59 (0.71)	147	2.89 (0.74)
NBHC seriousness scale	53	2.94 (0.75)	20	3.10 (0.55)
NBHC frequency of aggression scale	114	3.8 (0.92)	56	2.96 (0.85)
NBHC uses aggression scale	21	2.24 (0.44)	14	2.54 (0.88)
NBHC fear of retaliation scale	155	2.65 (0.74)	108	2.50 (0.70)

Higher mean scores indicate higher incidence of contributing factors of negative behaviors, seriousness of negative behaviors, frequency, use of aggression, and fear of retaliation (individual scores range from 1–5).
